# The effect of various subject characteristics on plantar pressure pattern

**DOI:** 10.1186/1757-1146-7-S1-A40

**Published:** 2014-04-08

**Authors:** Noël LW Keijsers, Niki M Stolwijk, Jan-Willem K Louwerens

**Affiliations:** 1 Department of Research, Sint Maartenskliniek, Nijmegen, the Netherlands; 2 Department of Orthopaedics, Sint Maartenskliniek, Nijmegen, the Netherlands

## Background

Plantar pressure is highly influenced by many factors such as walking velocity, body weight, and age. The impact of these subject characteristics on plantar pressure is usually studied separately. However, many of these factors are interact with each other; for example walking velocity is negatively correlated with body weight and age. The purpose of this study is to investigate the effect of several subject characteristics in relation to plantar pressure pattern for a large group of subjects.

## Materials and methods

Plantar pressure measurements of 589 subjects were used in this study. All subjects walked barefoot over a Foot Scan pressure plate (Rsscan International, Olen, Belgium) mounted on top of a force plate (Kistler Instruments, Switzerland) at their preferred walking speed. A total of five trials per foot were measured using a 3-step-protocol. Plantar pressure data was analyzed for each pixel by using the normalization method of Keijsers et. al. [1]. For each pixel, a multiple step forward linear regression analysis was used with mean pressure as dependent variable and the following subject characteristics as independent variables: body weight, contact time, age, body length, sex, foot progression angle, foot length, foot width, and side. Finally, the correlation coefficient of the full model for each pixel was calculated.

## Results

**Figure 1:**
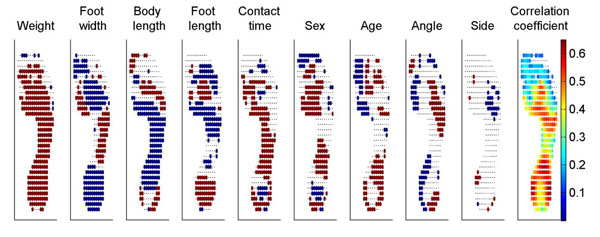
Red indicates an increase in pressure (sex: man; side: left) and blue a decrease in pressure with increasing subject characteristic.

The subject characteristics varied largely between subjects. The influence of each factor on the pressure of each pixel is shown in Figure 1. Body weight was the most important factor and was selected as parameter in 80.0% of the pixels. Body weight and walking velocity mainly have a positive effect on plantar pressure, whereas body length has a negative effect. The pressure under the heel, midfoot and distal part of the forefoot showed the highest correlation coefficient values with subject characteristics.

## Conclusions

Subject characteristics and especially the body weight and foot size play an important role in plantar pressure. Multiple regression analysis or adding subject characteristics as covariates is recommended when differences in plantar pressure between groups are studied.
